# How reliable is MRI in diagnosing cartilaginous lesions in patients with first and recurrent lateral patellar dislocations?

**DOI:** 10.1186/1471-2474-11-149

**Published:** 2010-07-05

**Authors:** Lars V von Engelhardt, Marthina Raddatz, Bertil Bouillon, Gunter Spahn, Andreas Dàvid, Patrick Haage, Thomas K Lichtinger

**Affiliations:** 1 Department of Trauma and Orthopedic Surgery, HELIOS-Klinikum Wuppertal, Heusnerstr. 40, 42283 Wuppertal, University of Witten/Herdecke, Germany; 2 Department of Trauma and Orthopedic Surgery, Medical Center Cologne-Merheim, Ostmerheimerstr. 200, 51109 Cologne, University of Witten/Herdecke, Germany; 3 Center of Trauma and Orthopedic Surgery Eisenach, Sophienstrasse 16, 99817 Eisenach, Germany; 4 Department of Diagnostic and Interventional Radiology, HELIOS-Klinikum Wuppertal, Heusnerstr. 40, 42283 Wuppertal, University of Witten/Herdecke, 42283 Wuppertal, Germany; 5 Department of Orthopedic and Trauma Surgery, St. Josef Hospital, Gudrunstrasse 56, Ruhr-University Bochum, 44791 Bochum, Germany

## Abstract

**Background:**

Lateral dislocation of the patella (LPD) leads to cartilaginous injuries, which have been reported to be associated with retropatellar complaints and the development of patellofemoral osteoarthritis. Therefore, the purpose of this study was to determine the reliability of MRI for cartilage diagnostics after a first and recurrent LPD.

**Methods:**

After an average of 4.7 days following an acute LPD, 40 patients (21 with first LPDs and 19 with recurrent LPDs) underwent standardized 1.5 Tesla MRI (sagittal T1-TSE, coronal STIR-TSE, transversal fat-suppressed PD-TSE, sagittal fat-suppressed PD-TSE). MRI grading was compared to arthroscopic assessment of the cartilage.

**Results:**

Sensitivities and positive predictive values for grade 3 and 4 lesions were markedly higher in the patient group with first LPDs compared to the group with recurrent LPDs. Similarly, intra- and inter-observer agreement yielded higher kappa values in patients with first LPDs compared to those with recurrent LPDs. All grade 4 lesions affecting the subchondral bone (osteochondral defects), such as a fissuring or erosion, were correctly assessed on MRI.

**Conclusions:**

This study demonstrated a comparatively good diagnostic performance for MRI in the evaluation of first and recurrent LPDs, and we therefore recommend MRI for the cartilage assessment after a LPD.

## Background

The consequences of patellar dislocation are cartilage injuries to the retropatellar joint and to the medial patellofemoral soft tissue complex with the prediction of subsequent instability [1-9]. Another factor that predisposes to recurrent LPD is trochlear dysplasia with insufficient trochlear depth, which is present in up to 85% of patients with recurrent patellar dislocation [10]. The incidence of chondral and osteochondral defects after first or recurrent LPD depends on the degree of lesions noted in previous studies. Based on a review of surgical studies, the frequencies of chondral and osteochondral lesions after LPD range between 32% and 96% [1,5-7]. Similarly, the frequency of cartilage injuries following LPD vary among several MRI studies, ranging from 30% to 75% [2,3,8]. Furthermore, a worsening of the articular cartilage was described at second-look arthroscopy approximately 1.5-2 years after the diagnosis of a LPD was made [11]. A 7-year non-operative follow-up study demonstrated high frequencies of full-thickness patellar (45%) and trochlear (31%) cartilaginous lesions, which were presumed to be a sign of developing osteoarthritis [9]. After an average follow-up of 13 years, Mäenpää *et al. *[4] diagnosed patellofemoral osteoarthritis in 22% of the patients, the highest frequency occurring in patients who underwent late surgery for patellofemoral pain or recurrent luxation. With respect to these data, accurate identification and appropriate treatment of cartilaginous lesions appears to be of special interest after patella dislocation. Thus, MR imaging, as a non-invasive method for cartilage assessment, could play an important role in the prevention of subsequent knee disability. This study was performed to investigate, whether MRI provides a reliable diagnostic performance for the assessment of the articular cartilage in patients with LPD. Therefore, cartilage diagnostics on standardized pre-operative MR images was compared to arthroscopic findings performed immediately after a first or recurrent LPD. To our knowledge, this study is the first of its kind to evaluate the diagnostic value of MRI for the cartilage assessment exclusively in a representative sample of patients with first and recurrent LPDs.

## Methods

### Subjects

**Figure 1:**
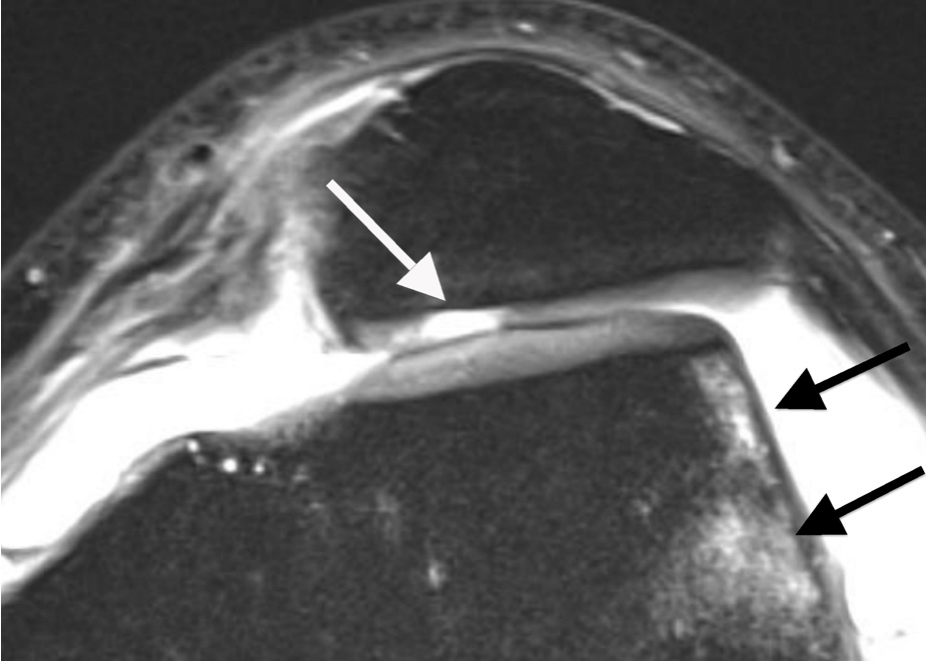
**Axial PD-weighted TSE MRI of a 28-year-old male with a first LPD**. MRI shows bone marrow edema involving the anterolateral femoral condyle (black arrow). At the central dome of the retropatellar articular surface, a full-thickness defect of the cartilage with denudation of the bone is visible. This finding is defined as a grade 4 disorder (white arrow).

This study was performed in accordance with the guidelines of our local Ethics Committee. MRI studies and arthroscopic surgery were performed for clinical indications. Furthermore, informed consent was given by all participants included in the study. Only patients who had a standardized MRI at our institution and subsequent arthroscopy soon after the LPD were included. In our emergency department, the diagnosis of acute LPD was confirmed by anamnesis and physical examination by an orthopedic surgeon. To be eligible for the study, patients had to have a firm clinical diagnosis of acute patella dislocation with a convincing history of dislocation, such as an acute twisting knee injury and/or a full giving way, as well as characteristic findings at physical examination. On follow-up MRI, all patients had typical findings suggestive of LPD, such as marrow edema involving both the anterolateral femoral condyle (Figure 1) and the inferomedial patella, and injury of the medial patellofemoral ligament. Of 40 patients (24 females and 16 males, mean age, 21.5 years) included between January 2006 and July 2009, 21 had first LPDs and 19 had histories of LPDs (1 patient with 1 LPD, 5 patients with 2 LPDs, 4 patients with 3 and 5 patients with > 3LPDs).

### MR imaging

**Figure 2:**
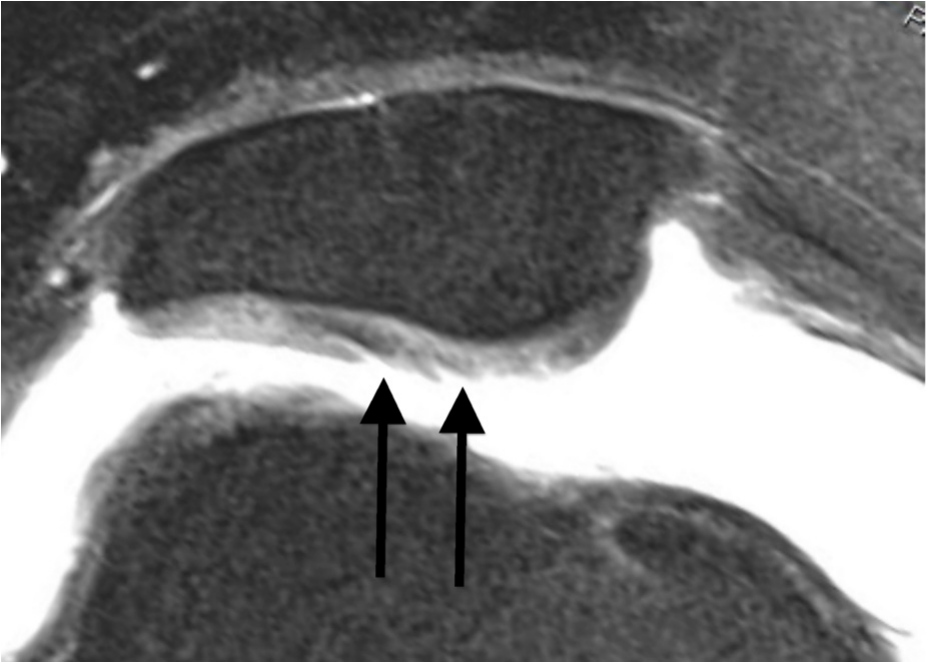
**Axial PD-weighted TSE MRI of a 29-year-old male with a recurrent LPD**. MRI shows a fibrillation, fissuring, or erosion composing < 50% of the cartilage thickness at the central dome and the lateral facet of the retropatellar articular surface (black arrow). This finding is defined as a grade 2 disorder.

**Figure 3:**
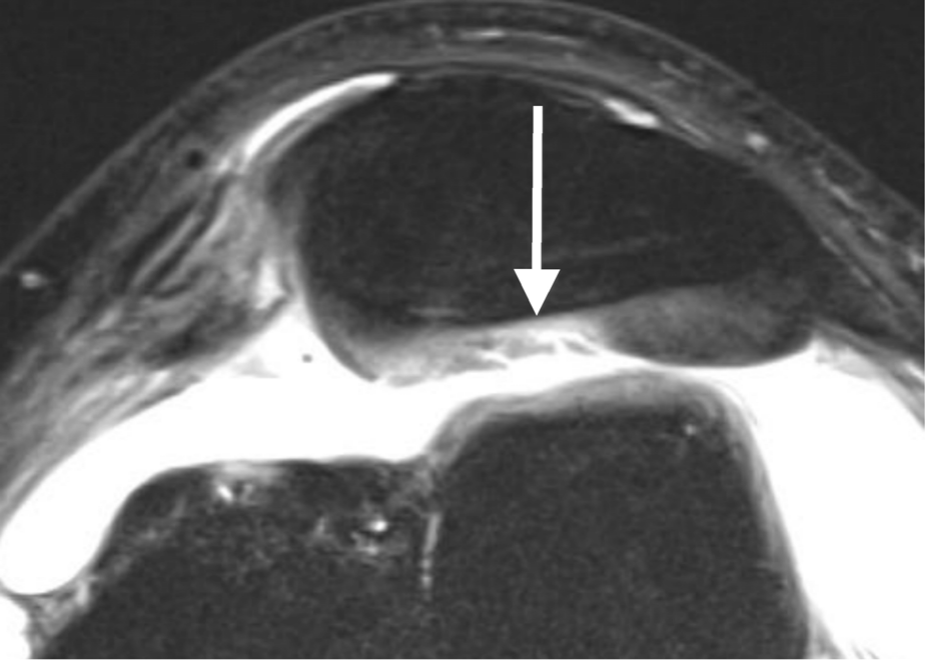
**Axial PD-weighted TSE MRI of a 29-year-old male with a first LPD**. A defect of > 50% of the retropatellar cartilage is depicted (white arrow). This finding is defined as a grade 3 disorder.

**Figure 4:**
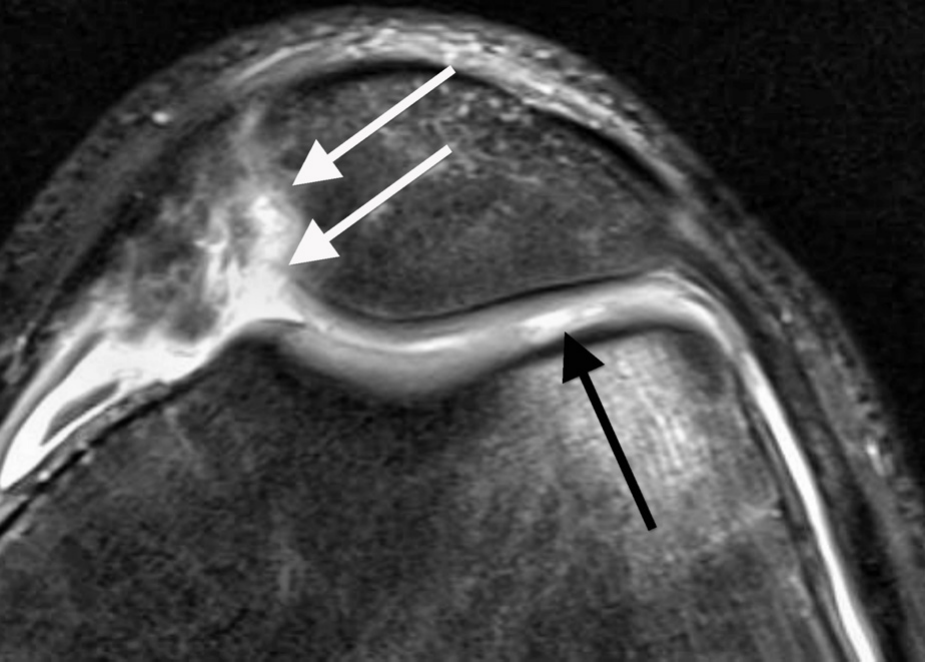
**Axial PD-weighted TSE MRI of a 15-year-old male with a recurrent LPD**. At the medial facet of the patella, a full-thickness defect of the cartilage (grade 4) with ulceration of the bone is demonstrated (white arrows). At the lateral femoral condyle, a cartilage composing > 50% of the cartilage thickness and showing a small ulceration to the subchondral bone is defined as a grade 3 lesion (black arrow).

**Figure 5:**
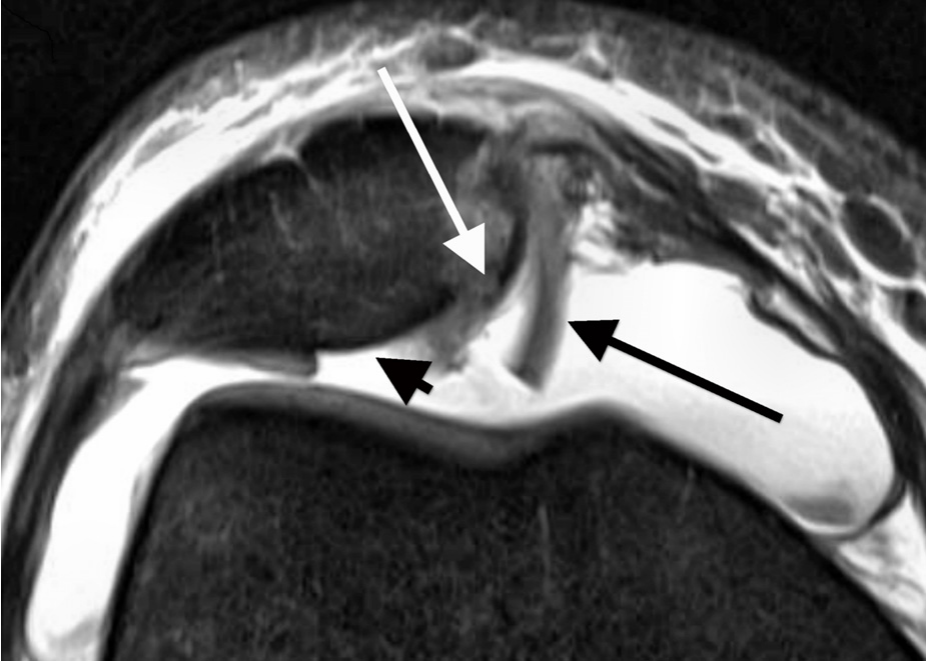
**Axial PD-weighted TSE MRI of a 41-year-old female with a first LPD**. At the medial facet of the patella, a full-thickness defect of the cartilage (grade 4) with fracture of the subchondral bone (long white arrow) and a free cartilage fragment (long black arrow) is visible. Furthermore, a full thickness cartilage defect at the central dome of the patella (short black arrows) is depicted. MR imaging of chondral or osteochondral fragments, cortical steps or bone destructions provides additional information when planning surgery, such as a refixation.

**Figure 6:**
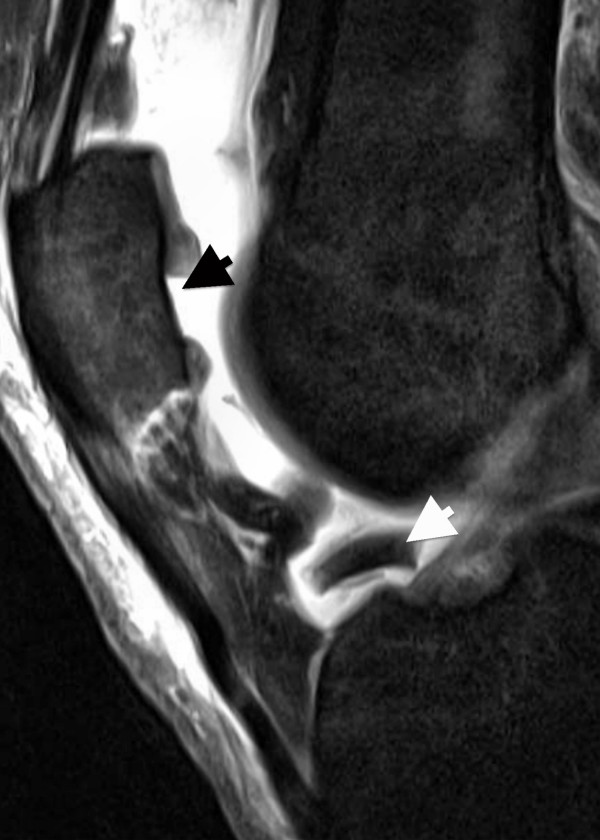
**Saggital PD-weighted TSE MRI of the same patient depicted in Figure 5**. A dislocated fragment lying in the intercondylar notch (short white arrow) corresponds to the full thickness cartilage defect at the central part of the patella (short black arrow).

The average time interval between acute LPD and MRI examination was 4.7 days (range, 0-19 days). All patients underwent standardized MR imaging on a 1.5-Tesla scanner (Siemens Magnetom Avanto syngo MR B 15, Erlangen, Germany) with a maximum gradient strength of 15 mT/m (rise time, 0.2 msec; slew rate, 150 mT/m/msec). A flexible synergy surface coil with two coil elements was used for imaging and was placed anterior and posterior to the knee. The following sequences were used in this study: a T1-weighted turbo spin-echo sequence (T1-TSE) in sagittal planes (field of view [FOV], 160 mm; matrix, 384; resolution, 0.4 mm × 0.4 mm × 0.6 mm; slices, 20; slice thickness, 4 mm; repetition time [TR], 461 ms; echo time [TE], 12 ms; flip angle [FA], 90°; acquisition time [AT], 4:29 min); a short tau inversion recovery sequence (STIR); TSE in coronal planes (FOV, 160 mm; matrix, 256; resolution, 0.8 mm × 0.6 mm × 4.0 mm; slices, 20; slice thickness, 4 mm; TR, 5100 ms; TE, 27 ms; FA, 160°; AT, 5:43 min); a transversal proton density (PD)-weighted TSE with fat suppression (FOV, 150 mm; matrix, 256; resolution, 0.6 mm × 0.6 mm × 3.0 mm; slices, 20; slice thickness, 3 mm; TR, 965 ms; TE, 26 ms; FA, 40°; AT, 4:09 min); and a PD-weighted TSE with fat suppression in sagittal planes (FOV, 160 mm; matrix, 256; resolution, 0.6 mm × 0.6 mm × 4.0 mm; slices, 20; slice thickness, 4 mm; TR, 951 ms; TE, 26 ms; FA, 40°; AT, 4:05 min). MR images were reviewed separately on a PACS workstation (ID.Read; Image Devices, Taunusstein, Germany) by two orthopedic surgeons experienced in diagnostics and the treatment of knee disorders (MR and LVvE). Retrospective MRI readings were performed in several sessions between November 2009 and January 2010. Both readers were blinded to any clinical data. The MR images were reviewed in alphabetical order, irrespective of the diagnosis of cartilaginous lesions, and first or recurrent LPD. During the MRI interpretation, which considered all MRI sequences, the readers were able to freely adjust image brightness, contrast, and zoom. To compare the MRI results to arthroscopic findings, the articular surface of the retropatellar joint was divided into the following six regions: medial facet of the patella; central dome; lateral facet; and medial, central and lateral trochlear grooves of the femoral condyle. Each cartilage surface was analyzed as a single entity. In order to perform a direct comparison between MRI and arthroscopy, we used a classification based on the macroscopic Outerbridge grading [12]. This MRI classification has been used in several previous studies evaluating MR imaging of the articular cartilage [13-15]. Grade 0 is defined as cartilage with a normal intrinsic signal and a normal surface contour on MR images. Cartilage with a smooth surface and the presence of signal heterogeneities with focal areas of hyper-intensity is defined as a grade 1 lesion. On MRI, a grade 2 disorder is characterized by a fibrillation, fissuring, or erosion composing < 50% of the thickness of the cartilage (Figure 2). Defects > 50% on MR imaging are defined as grade 3 and occur with or without small bone ulcerations (Figures 3 and 4). Extended full-thickness lesions with denudation of the bone are defined as grade 4 (Figures 1, 5, and 6). Grade 4 cartilage defects were also noticed with osteochondral injuries, such as ulcerations (Figure 4) or fissuring (Figure 5) of the subchondral bone. In cases of multiple cartilage defects within one of the six articular surfaces, only the highest grade of cartilage damage was documented.

### Arthroscopy

The average period between MRI and arthroscopy was 16 days (range, 1-135 days). Arthroscopic grading of cartilage disorders was performed by six orthopedic surgeons experienced in knee surgery. At the time of arthroscopy, the MR images were available to the surgeon, whereas the MRI grading of the hyaline cartilage was not available. Surgery was performed using the standard antero-medial and antero-lateral portals. Each knee compartment was inspected thoroughly and palpated using a blunt hook. Arthroscopic findings of the cartilage were classified as grades 0-4, according to the system of Outerbridge [12]. Cartilage damage was treated in the same session with abrasion (16 patients), resection of free chondral or osteochondral fragments (12 patients), refixation of chondral or osteochondral fragments (5 patients), and drilling (3 patients). Furthermore, loose bodies and hemarthroses were removed in the same session. Other injuries, such as meniscal lesions (three patients) and anterior cruciate ligament tears (one patient), were seldom noticed.

### Statistical analyses

In both patient groups, sensitivities, specificities, and positive and negative predictive values of MRI were calculated for each grade of cartilaginous disease. Diagnostic values were calculated using JavaStat http://statpages.org/ctab2x2.html. The kappa statistic was used to measure inter-observer agreement. The software program, PASW statistics 18 (SPSS, Inc., Chicago, IL, USA), was used for the data transformation. Weighted kappa values for multiple categories and their 95% confidence intervals were calculated using the web-based kappa Calculator for Clinical Research http://faculty.vassar.edu/lowry/kappa.html. According to Landis and Koch, a kappa value of < 0.20 indicates poor agreement, 0.21-0.40 indicates fair agreement, 0.41-0.60 indicates moderate agreement, 0.61-0.80 indicates good agreement, and 0.81-1.0 indicates very good agreement [16].

## Results

There were only 3 patients (8%) without any cartilage disease noted during arthroscopic assessment. The distribution of cartilaginous lesions within the patellofemoral joint is depicted in Table 1. The MRI grading of both reviewers were compared to arthroscopic findings (Table 2), showing an exact agreement in 78% (186 of 240) and 76% (182 of 240) of the joint surfaces. As presented in Table 3, intra- and inter-observer agreement differed markedly between the patient cohorts. Thus, moderate-to-good kappa values were obtained in patients with recurrent LPD, whereas good-to-very good values were yielded in patients with first LPD.

**Table 1:** Distribution of cartilage disorders within the patellofemoral joint during arthroscopic assessment

	Cartilage lesions in all patients with acute LPD (1^st ^LPD/recurrent LPD)			
	**Grade 4**	**Grade 3**	**Grade 2**	**Grade 1**

Medial facet	17 (12/5)	7 (1/6)	10 (5/5)	1 (0/1)
Central dome	4 (3/1)	4 (3/1)	6 (1/5)	2 (1/1)
Lateral facet	1 (0/1)	1 (1/0)	0 (0/0)	1 (0/1)
Medial trochlear groove	0 (0/0)	0 (0/0)	4 (2/2)	2 (1/1)
Central trochlear groove	1 (1/0)	0 (0/0)	4 (2/2)	1 (0/1)
Lateral trochlear groove	5 (2/3)	2 (0/2)	7 (5/2)	2 (0/2)

**Table 2:** Comparison between both readers with respect to MRI grading and arthroscopic grading of the cartilage

	MRI grading of both MRI readers (reader 1/reader 2)				
**Arthroscopic grading**	**Grade 0**	**Grade 1**	**Grade 2**	**Grade 3**	**Grade 4**

Grade 0	143/138	6/10	8/8	0/1	1/1
Grade 1	8/7	1/1	0/1	0/0	0/0
Grade 2	15/13	0/0	11/14	3/2	2/2
Grade 3	1/2	0/0	3/3	8/7	2/2
Grade 4	1/1	0/0	3/1	3/2	23/22

**Table 3:** Weighted kappa values and 95% confidence intervals for both MRI readers, and inter- and intra-observer agreement in all patients with LPD, in patients with a first LPD, and in patients with a recurrent LPD

	Weighted kappa scores^†^		
	**All patients**	**First LPD**	**Recurrent LPD**

Reader 1 vs. Reader 2	0.75 (0.68-0.83)	0.82 (0.74-0.91)	0.67 (0.55-0.80)
AC* vs. reader 1	0.73 (0.65-0.80)	0.83 (0.75-0.91)	0.61 (0.48-0.74)
AC* vs. reader 2	0.70 (0.62-0.78)	0.79 (0.70-0.88)	0.60 (0.47-0.73)

^† ^< 0.20 = poor, 0.21-0.40 = fair, 0.41-60 = moderate, 0.61-0.80 = good, 0.81-1.0 = very good

* AC = arthroscopic findings

Both patient groups were assessed separately for the diagnostic values of each grade of cartilage disease (Table 4). For grade 3 and 4 lesions, the patient group with first LPDs had markedly higher sensitivities and positive predictive values than those with recurrent LPDs. Of 12 patients with osteochondral injuries at the medial patella, 9 had their first LPD. These lesions were all correctly assessed at MRI as grade 4 cartilage defects with ulceration (Figure 4) or fissuring (Figure 5) of the subchondral bone.

**Table 4:** Diagnostic values of MRI readings (reader 1/reader 2) for each grade of cartilaginous lesion in patients with first and recurrent LPDs

		Sensitivity [%]	Specificity [%]	Positive predictive value [%]	Negative predictive value [%]
**Grade 4**	First LPD	89/83	99/100	94/100	98/97
	Recurrent LPD	70/70	96/95	64/58	97/97

**Grade 3**	First LPD	83/60	98/98	63/60	99/98
	Recurrent LPD	60/44	97/97	67/57	96/95

**Grade 2**	First LPD	33/40	97/96	63/55	92/92
	Recurrent LPD	38/50	91/91	40/47	90/92

**Grade 1**	First LPD	0/0†	98/96	0/0†	98/98
	Recurrent LPD	14/14	98/97	20/11	97/97

† None of the patients with a first LPD had a grade 1 lesion at arthroscopy. Diagnostic values were therefore not ascertainable.

## Discussion

During LPD, the medial facet of the patella impacts against the lateral femoral condyle, which can lead to corresponding injuries of the articular surface. Because dislocation is usually transient, the patella recoils back and the corresponding articular surfaces can sustain injury again. This leads to a high incidence and typical locations of cartilaginous defects [1-3,5,6,8]. In agreement with previous studies on LPD, cartilaginous lesions were predominately noted at the medial facet of the patella (55%) and the lateral femoral condyle (25%; Table 1) [1,5,6]. According to clinical follow-up studies, as well as experimental studies, chondral lesions may increase the risk of subsequent patellofemoral joint symptoms and osteoarthritis [4,9,11,17]. Therefore, accurate identification and appropriate treatment of cartilaginous lesions following LPD play an important role in minimizing knee disability.

Based on the literature, the MRI sequence best suited for cartilage diagnostics is still under debate [18-21]. Cartilage-specific sequences, such as spoiled gradient-recalled echo and fast low-angle shot sequences, provide a high spatial resolution and have therefore been described as being useful in segmenting techniques for quantitative cartilage studies. The disadvantages of these sequences are a high sensitivity to susceptibility artifacts and a limited visualization of the subchondral bone, menisci, and ligaments [19,22]. Most experience and good results for the detection of cartilage and subchondral bone disorders were gathered with T2-, intermediate- and PD-weighted fast spin echo sequences [19,20,23-25]. In the current study, we used fat-suppressed PD-weighted fast spin-echo sequences with a 3-mm slice thickness in transverse planes and a 4-mm slice thickness in sagittal planes. In previous reports on comparable sequences with and without fat suppression, 1.5 Tesla MRI was described to depict the articular cartilage with an accuracy comparable to that of several cartilage-specific sequence protocols [18,20,21,24]. Similar results were noticed for T2- and intermediate-weighted fast spin echo sequences, which also yielded comparable results to those of other cartilage-specific sequence protocols [23,26,27]. However, our study demonstrated relatively good inter- and intra-observer agreement (Table 3) in comparison to previous MRI studies on cartilage grading, in which kappa values ranged from 0.60-0.93 [13,21,28,29].

In patients with first and recurrent LPDs, the diagnostic performance of MRI for cartilaginous lesions was evaluated for each grade of cartilage disease (Table 4). At each grade, the specificities and negative predictive values were relatively high, giving MRI a certain importance for the exclusion of cartilaginous lesions. In agreement with the literature, the sensitivities for the detection of grade 1 and 2 lesions were poor (Tables 2 and 4) [13,18,30]. Thus, reliable MRI differentiation of superficial erosions or fibrillations from intact cartilage appears difficult after LPD.

Regarding grade 3 and 4 lesions, patients with first LPD showed markedly higher diagnostic values compared to those with recurrent dislocation. Reader 1's sensitivity and positive predictive value for grade 4 lesions were 89% and 94% in patients with first LPDs, but only 70% and 64% after LPDs, respectively. Likewise, reader 1's diagnostic values for grade 3 lesions were higher in patients with a first LPD compared to those with a recurrent LPD (Table 4). Similar tendencies existed in reader 2's sensitivities and positive predictive values. Regarding the positive predictive values in patients with recurrent LPDs, the probability that the MRI finding of a grade 3 and 4 defect corresponds exactly to the arthroscopic finding was between 57% and 64%. Therefore, the value of MRI for a detailed assessment and grading of the cartilage should not be overestimated, especially after recurrent LPDs. Likewise, the kappa values for the intra- and inter-observer agreements yielded markedly better results in patients with first LPDs compared to those with recurrent LPDs (Table 3). Regarding the kappa values (Table 3) and the diagnostic values for grade 3 and 4 lesions (Table 4), we assume that MRI is more reliable for the diagnosis of cartilaginous defects in patients with first LPDs, whereas the diagnostic performance is limited after recurrent LPDs.

Better diagnostic values in patients with first LPDs could be explained in part by the higher severity of trauma. Thus, as reported by others, severe cartilaginous lesions with ulceration or fissuring of the subchondral bone were more frequent in patients with first LPDs (48%) compared to patients with recurrent LPDs (16%; Table 1) [1,3,5,7,9,31]. In this context, it has to be mentioned that arthroscopically-detected osteochondral lesions occurring after LPDs were identified with pre-operative x-ray in 29% and 60% of the cases [31,32]. Thus, correct identification of osteochondral lesions appears to be limited on standard radiographs. In contrast, all osteochondral defects in our study were correctly assessed at MR imaging as grade 4 cartilage lesions with ulceration (Figure 4) or fissuring (Figure 5) of the subchondral bone. In addition to a role for the detection of osteochondral lesions, MRI could be of practical assistance in planning the surgery. In our patient cohort, visualization of difficulties for a refixation of osteochondral fragments, such as cortical steps (Figure 5) and bone destructions (Figure 4), as well as the visualization of intra-articular loose bodies (Figures 5 and 6), provided additional information before surgery. Therefore, we suggest that MRI is an excellent diagnostic tool for osteochondral lesions in patients with LPD.

A limitation of this study was the use of the Outerbridge classification for cartilage assessment. Recent reports describe quantitative, semi-quantitative, and whole organ approaches for MRI assessment of the cartilage as reliable scoring and research tools, especially in patients with osteoarthritis [22,29]. Furthermore, the use of arthroscopic grading as a reference standard should be regarded with caution. In the literature, inter-observer agreement at arthroscopy demonstrates sufficient reproducibility [33], but poor results for cartilage grading [34]. On the other hand, a study by Bachmann *et al. *[35] yielded an exact agreement between arthroscopic and histopathologic grading in 287 of 300 cases. Thus, the arthroscopic method is a valuable tool in clinical research to score chondropathies, even if inspection and palpation with the hook probe cannot detect all changes of the cartilage as a histomorphologic evaluation.

## Conclusions

In comparison to studies of other knee disorders, MRI yielded a relatively good performance in patients with LPD. For the diagnosis of grade 3 and 4 cartilaginous defects, diagnostic values were limited in patients with recurrent LPDs, whereas markedly better results were assessed after first LPDs. For osteochondral defects, MRI was a reliable diagnostic tool, and of practical assistance when performing surgery. Therefore, we recommend MRI for the diagnosis of chondral and osteochondral defects after LPD. Accurate MRI diagnosis of cartilage defects could help minimize knee disability in the future when followed by appropriate treatment.

## Competing interests

The authors declare that they have no competing interests.

## Authors' contributions

LVvE, GS, AD, BB, PH, and TKL conceived and designed the study. LVvE, MR, PH, and AD were involved in the execution of the study. In addition, LVvE and MR performed MRI grading and LVvE performed the statistical analysis. LVvE, GS, MR, PH, AD, BB, and TKL were involved in the writing and proofreading of this manuscript. All authors read and approved the final manuscript.

## Pre-publication history

The pre-publication history for this paper can be accessed here:

http://www.biomedcentral.com/1471-2474/11/149/prepub
